# The dynamic nature of frailty in metastatic spine disease patients

**DOI:** 10.1007/s11060-025-05213-8

**Published:** 2025-09-09

**Authors:** Oludotun Ogunsola, Edward S. Harake, Sean Smith, Michael Albdewi, Varun Kathawate, Sebele Ogunsola, William Jackson, Joseph Evans, Vikram Chakravarthy, Nicholas Szerlip

**Affiliations:** 1 Department of Neurosurgery, University of Michigan, Ann Arbor, MI USA; 2 University of Michigan Medical School, Ann Abor, MI USA; 3 Department of Physical Medicine and Rehabilitation, University of Michigan, Ann Arbor, MI USA; 4 Perelman School of Medicine, University of Pennsylvania, Philadelphia, PA USA; 5 Western Michigan University Homer Stryker MD School of Medicine, Kalamazoo, MI USA; 6 Department of Radiation Oncology, University of Michigan, Ann Arbor, MI USA; 7 The Ohio State University Wexner Medical Center, Columbus, OH USA

**Keywords:** Frailty, Intervention, Metastatic spine disease, Patient management, Survival

## Abstract

**Purpose:**

Frailty measures are critical for predicting outcomes in metastatic spine disease (MSD) patients. This study aimed to evaluate frailty measures throughout the disease process.

**Methods:**

This retrospective analysis measured frailty in MSD patients at multiple time points using a modified Metastatic Spinal Tumor Frailty Index (MSTFI). Scores were 0: “not frail,” 1: “mild”, 2: “moderate, and ≥ 3: “severe.” Measurements were taken at cancer diagnosis, spine metastasis (SM) diagnosis, and 4-month intervals up to 2-years. The change in frailty distribution was described at the general cohort and patient levels. Two-year survival was assessed from baseline frailty status at SM diagnosis.

**Results:**

This study included 465 patients with an average age of 62.3 years (± 12.7), 33.8% female. Prostate cancer was most common (20.9%), followed by renal cell carcinoma (15.3%), non-small cell lung cancer (NSCLC, 13.5%), and others. Frailty changed dynamically, most significantly early in the disease. Initially, 79.4% were not frail; this dropped to 60.1% at SM diagnosis and to 42.7% at 4 months. Patients with short-term (4 month) data showed rapid frailty progression, with 57% becoming moderately to severely frail (*p* = 0.01), whereas 45% with long-term (24 months) data remained non-frail (*p* < 0.0001). Contributing factors included anemia (32.7%), electrolyte abnormalities (16.9%), and malnutrition (11.4%). Histological classification influenced frailty. Higher frailty scores at metastatic diagnosis correlated with worse 2-year survival outcomes (p: <0.001–0.04), underscoring frailty’s prognostic significance.

**Conclusion:**

Frailty is dynamic, with a potential early intervention point to maintain or reverse it. Further research is needed to assess which frailty measures are most dynamic and amenable to intervention.

## Introduction

Frailty is a multidimensional syndrome characterized by a decline in physiological reserves, leading to increased vulnerability to adverse health outcomes. In medicine, frailty has been extensively utilized to predict clinical outcomes across various contexts, including emergency room triage, surgical triage, and internal medicine. Frailty indices have been shown to predict hospital readmissions, postoperative complications, and overall mortality in both acute and chronic care settings [1–7]. Frailty indices have been explored in cardiovascular disease and orthopedic surgery as well, emphasizing their broad applicability in risk stratification and treatment planning. Most relevant in metastatic spine disease (MSD), frailty is emerging as a critical predictor of functional outcomes and survival, emphasizing the importance of integrating frailty assessments into oncologic care and surgical decision-making [8–12].

For patients with metastatic spinal tumors, frailty has been measured using various approaches, including functional measures such as grip strength, gait speed, and balance tests, which directly assess physical performance [13]. Self-reported metrics, including validated patient-reported outcome measures (PROMs) like the EQ-5D and PROMIS Physical Function, allow patients to report their perceived health status and functional limitations [14–16]. Chart-based methods, such as the Metastatic Spinal Tumor Frailty Index (MSTFI), rely on retrospective review of patients’ status and are particularly useful for assessing frailty in large cohorts where direct physical assessments may be impractical [17, 18]. Each method offers unique insights into patient health, with functional tests providing direct assessments, self-reported metrics reflecting patient perspectives, and chart-based methods facilitating population-level analysis [19].

As frailty is increasingly recognized as a significant predictor of adverse outcomes across medical specialties, in MSD, it may provide an assessment of patient vulnerability and guide surgical treatment decisions. Despite its prognostic utility, interventions targeting frailty remain limited in routine clinical practice. This gap persists partly due to the complex, multifactorial nature of frailty and the challenges in standardizing effective intervention strategies [20–23]. There are ongoing clinical trials exploring frailty-targeted interventions, particularly in populations with chronic diseases and cancer, aiming to improve patient resilience and outcomes. Investigating whether frailty can be modified in MSD patients is crucial, as it could shift frailty from a passive risk marker to an actionable intervention, ultimately enhancing treatment tolerability and overall prognosis in this vulnerable patient population [24, 25].

This study explored the longitudinal changes of frailty among patients with MSD undergoing stereotactic body radiation therapy (SBRT), examining factors that influence its progression and their implications for clinical practice. Through a systematic analysis of patient data, we investigated the trajectory of frailty and identified key predictors of frailty transitions.

## Methods

### Study design and participant demographics

A retrospective analysis, conducted and reported in accordance with STROBE guidelines, involved a cohort of MSD patients who were followed at a tertiary academic hospital from 2012 to 2022 after receiving SBRT to a spinal lesion. Approval for this study was obtained from the University of Michigan (Ann Arbor, Michigan, 48109, USA) Institutional Review Board (ID HUM00139855); patient consent was waived. The primary outcome was patients’ frailty status at initial presentation and its trend over time during oncologic management. Pertinent demographic information and medical history were collected including the tumor stage at initial cancer diagnosis, histologic characterization of the metastatic lesion(s), Bilsky grade of the lesion(s), treated vertebral levels, and type of treatment (i.e., hormone, cytotoxic, immunotherapy, and targeted therapy).

## Data collection

Frailty was assessed using a modified MSTFI, developed by De la Garza Ramos et al., which includes the sum of 9 dichotomous variables: anemia, chronic lung disease, coagulopathy, electrolyte abnormalities, pulmonary circulation disorders (PCD), renal failure, malnutrition, emergent/urgent hospital admission, and the surgical approach. Each variable was worth 1 point, except PCD, which was 2 points. Our version, termed MSTFI-7, excluded hospital admission and surgical approach, focusing only on manageable comorbidities. The presence of each variable was determined via retrospective review of each patients’ electronic health record. To standardize the collection process, the following criteria were used: anemia, coagulopathy, renal failure, and electrolyte abnormalities were determined via normal laboratory value ranges. Chronic lung disease and PCD were determined via explicit mention or diagnosis in clinical notes. Malnutrition was assessed in accordance with the Global Leadership Initiative on Malnutrition criteria, by using a combination of albumin levels and clinical note mention of significant weight loss [26]. Scores were determined by adding up the variable counts and then classified as follows: 0 = “Not frail”, 1 = “Mild”, 2 = “Moderate”, and > = 3 = “Severe.”

To evaluate general cohort frailty dynamics, MSTFI-7 scores were calculated at: (1) initial cancer diagnosis, (2) diagnosis of spinal metastasis (SM), and (3) every 4 months for 2-years post SM diagnosis. This allowed the visualization of frailty evolution over time. Dynamic change was defined as overall progression or improvement in frailty status over time. Since not all patients had available data for all 2-years of post-diagnostic timepoints, the mortality rate within each time interval was collected to assess the impact of patient mortality on cohort size over time. To then approximate patient-level frailty changes over time, patients were grouped by available timepoint data: those with only 4 months of timepoints, those with exactly 1 year of timepoints, and those with all 2 years of data post-diagnosis. We documented intra-group frailty status distribution and transitions between 3 groups overtime (i.e., Not frail, Mild frailty, Moderate-severe frailty).

Contributing factors to patients’ frailty scores were also evaluated. First, the relative contributions of each MSTFI-7 component on overall frailty were descriptively analyzed by assessing the proportion of patients with each given condition at each recorded time point. Next, mixed-effect modeling was implemented to determine patient variables with a strong relationship to frailty score. Mixed-effect models were used for their strong utility in longitudinal, repeated-measurement studies, as they account for both fixed and random effect variables. While the former pertained to variables with constant effect across the cohort (e.g., effect of treatment on all patients), the latter addressed individual-level variations. In this study, incorporated random-effect variables consisted of each individual patient while fixed-effect variables included demographic information (i.e., age), histologic group, and treatment approach. Reported main-effect coefficients indicated the average change in frailty score for each unit increase or positive presence of an independent variable. In interaction terms (e.g., histologic group: 4-month only group), the coefficient indicated the relative change to the baseline coefficient of the histologic group. The combined effect is the difference of the two coefficients reported in the Results section.

## Survival outcomes

Using a Kaplan-Meier survival analysis, a time-based probability of 2-year survival was calculated for patients across 3 categories: Not frail, Mild frailty, and Moderate-severe frailty. All survival curves began from the initial diagnosis of spinal metastasis. The probabilities among groups were then compared for significant differences.

### Statistical analysis

Data analysis was performed with Python using open-source analytical libraries. The rates of categorical variables were compared with Chi-squared analysis, and continuous variables were compared with two-sample independent *t*-tests (2 groups) and ANOVA (> = 3 groups). Notably, missing data for a particular variable were excluded without imputation in both our dataset summarization statistics as well as mixed effect modelling in order to obtain more accurate results and reduce bias. To descriptively analyze frailty trends over time, intragroup analyses based on available time points were not controlled for baseline frailty scores. Survival curves were compared using log rank tests. All statistical analyses were conducted with an alpha value of 0.05. Given the relatively small number of pairwise comparisons across histologic groups, and to reduce the risk of false negatives, we did not apply further *P* value corrections.

## Results

### Demographics and medical history

The cohort consisted of 465 patients (average age: 62.3 +/- 12.7 years, 33.8% female (Table 1). The most common cancer types were prostate (20.9%), renal cell carcinoma (RCC, 15.3%), non-small-cell lung cancer (NSCLC) (13.5%), and others (29.0%). Most patients were treated at the thoracic (61.1%) and lumbar (37%) levels, with 61.7% having Bilsky grade 0.

**Fig. 1:**
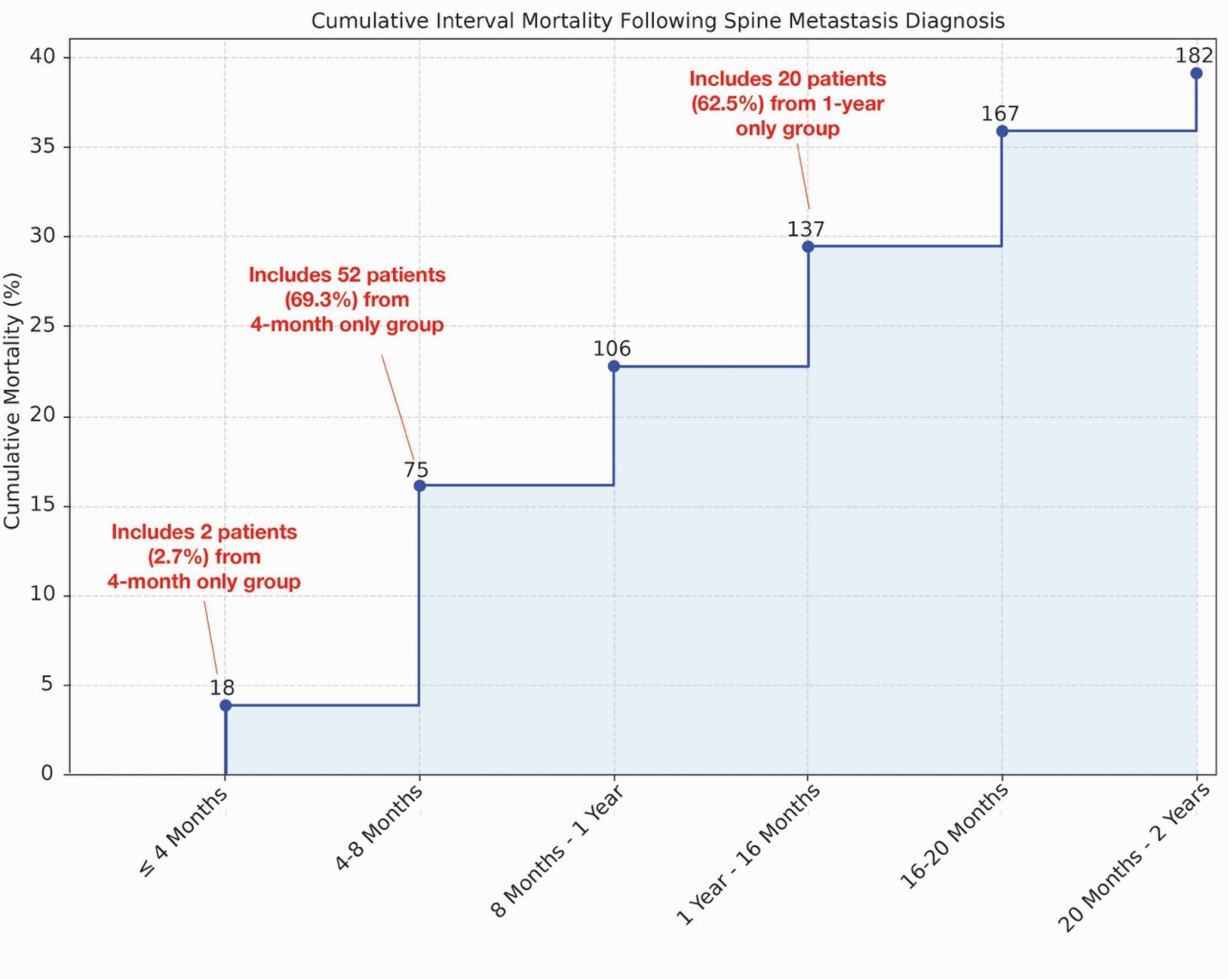
Cumulative interval mortality rates following spine metastases diagnosis. The figure depicts the number and proportion of patients who died within each 4-month interval following spinal metastatic diagnosis. By 2 years the cumulative mortality rate within the general cohort was roughly 40%. Notably, nearly 70% of patients with only 4-months of follow-up timepoints died by the 4–8-month interval, and about 63% of 1-year only patients died within the 12–16-month interval. This highlights that the limited available follow-up in these cohorts was largely driven by mortality rather than loss of follow-up

Patients were grouped by available time-points post-SM diagnosis: 75 with only 4 months, 32 with 1 year, and 164 with all 2 years. The variability (*P* < 0.001) in available time points was proportional to survival time following SM diagnosis: 179 (+/- 114) days in the 4-month group, 418 (+/−63) days in the 1-year group, and 1,265 (+/- 866) days in the 2-year group. Notably, limited time points within each group were primarily driven by mortality as opposed to loss of follow-up. Over 70% of patients in the 4-month only group died prior to the 8-month follow up, and nearly 63% of 1-year only patients died before 16-month follow-up (Fig. 1).

Patients with 2-year data had significantly fewer Bilsky grade 1 epidural tumors (17% vs. 29.3–37.5%; *P* = 0.01) and a nonsignificant but higher rate of Bilsky grade 2 epidural tumors (15.8% vs. 3.1–9.3%; *P* = 0.08). (Table 1) Patients with only 4-months of data had a significantly lower representation of prostate cancer compared to 2-year cohort (8% vs. 29.3%, *P* < 0.01), but a significantly higher rate of melanoma (13.3% vs. 3%; *P* = 0.01). Those with only 1-year of data had a higher rate of sarcoma relative to 4-month patients (21.9% vs. 4%, *P* = 0.01) (Table 1).

**Table 1:** Summarization of demographics, radiologic findings, and histology

Characteristics	General	Only 4-mo follow-up	Only 1-y follow-up	2-y follow-up	*P* overall	*P* 4-mo vs. 1-y	*P* 4-mo vs. 2-y	*P* 1-y vs. 2-y
**Total**	465	75	32	164				
**Sex**								
Male	308 (66.2%)	50 (66.7%)	24 (75%)	107 (65.2%)	0.57	0.53	0.95	0.39
Female	157 (33.8%)	25 (33.3%)	8 (25%)	57 (34.8%)				
**Age**	62.3$\:\pm\:$12.7	61.5$\:\pm\:$11.7	62.9$\:\pm\:$14.3	62$\:\pm\:\:$11.9	0.87	0.62	0.79	0.71
**BMI**	27.7$\:\pm\:$6.1	27.6$\:\pm\:$6.7	27.6$\:\pm\:$4.6	28.7$\:\pm\:$6.8	0.5	0.97	0.31	0.45
**Treated level**								
Cervical	71 (15.3%)	14 (18.7%)	7 (21.9%)	24 (14.6%)	0.51	0.91	0.55	0.45
Thoracic	284 (61.1%)	51 (68%)	20 (62.5%)	99 (60.4%)	0.53	0.74	0.32	0.98
Lumbar	172 (37%)	23 (30.7%)	7 (21.9%)	64 (39.0%)	0.12	0.49	0.27	0.1
Sacral	43 (9.2%)	4 (5.3%)	2 (6.3%)	19 (11.6%)	0.25	1	0.2	0.56
**Bilsky grade**								
0	287 (61.7%)	46 (61.3%)	19 (59.4%)	110 (67.1%)	0.58	1	0.47	0.52
1	109 (23.4%)	22 (29.3%)	12 (37.5%)	28 (17.1%)	**0.01**	0.55	**0.046**	**0.02**
2	69 (14.8%)	7 (9.3%)	1 (3.1%)	26 (15.8%)	0.09	0.47	0.25	0.1
**Histology**								
Prostate	97 (20.9%)	6 (8%)	6 (18.8%)	48 (29.3%)	**0.001**	0.2	**0.001**	0.32
RCC	71 (15.3%)	11 (14.7%)	2 (6.3%)	22 (13.4%)	0.47	0.37	0.95	0.4
NSCLC	63 (13.5%)	15 (20%)	5 (15.6%)	20 (12.2%)	0.28	0.79	0.17	0.81
Sarcoma	41 (8.8%)	3 (4%)	7 (21.9%)	15 (9.2%)	**0.01**	**0.01**	0.26	0.08
Breast	33 (7.1%)	3 (4%)	0	14 (8.5%)	0.12	0.61	0.32	0.18
Melanoma	25 (5.4%)	10 (13.3%)	2 (6.3%)	5 (3.0%)	**0.01**	0.47	**0.01**	0.71
Other	135 (29.0%)	27 (36%)	10 (31.3%)	40 (24.4%)	0.17	0.8	0.09	0.55
Survival time from diagnosis*	654.3$\:\pm\:$690.1	178.5$\:\pm\:\:$114	417.8$\:\pm\:$63.1	1264.8$\:\pm\:\:$866	**< 0.001**	**< 0.001**	**< 0.001**	**< 0.001**

*Days

Abbreviations: *BMI* Body mass index, *NSCLC N*on-small cell lung cancer, *mo* Month(s), *P* P value, *RCC,* Renal cell carcinoma,*y* year(s). Bold indicates statistical significance

Preoperative and postoperative variables are stratified (above) by available follow-up data from metastatic diagnosis to group similar patients and approximate an individual-level analysis. These groups exhibited significantly different survival times, with 4-month only patients surviving roughly seven times less than patients with all 2-years of data. Preoperatively, demographics were similar among all groups, but the 2-year patients exhibited significantly fewer incidents of Bilsky Grade 1 tumors. Postoperatively, histologic classification differed by group: 4-month only patients had significantly fewer prostate cancer diagnoses and greater melanoma diagnoses, while 1-year patients had more sarcoma diagnoses.

### Frailty status: dramatic change over time

**Fig. 2:**
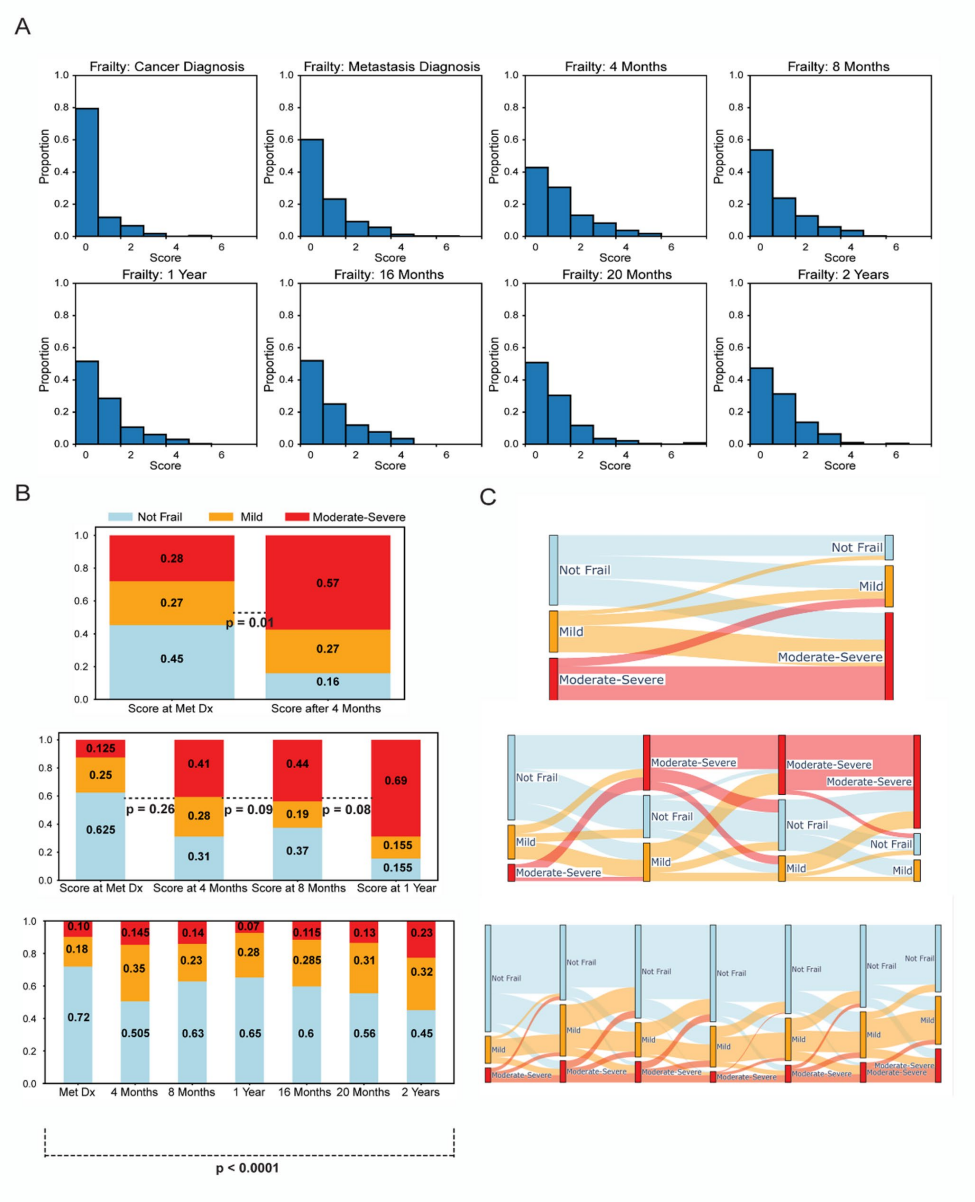
Dynamic changes in frailty status. **A** The change in frailty status overtime in the total cohort was assessed using the MSTFI-7 score (0 = Not Frail, 1 = Mild Frailty, 2 = Moderate Frailty, and > = 3 = Severe Frailty). The greatest increases in the number of frail patients occurred progressively between the initial cancer diagnosis and the four-month follow-up from discovery of SM. **B** Frailty status was then evaluated at the individual level by comparing the rates of different frailty severities overtime and (**C**) characterizing the transitions between severity classes. Patients with fewer than 2 years of post-diagnostic data presented frailer, exhibited fast progression of frailty, and showed nearly negligible improvement in those who reached severe frailty status. Patients with all 2 years of data showed a slower, more stable progression of frailty overtime

Frailty revealed dynamic changes, with an early and significant increase after SM diagnosis. Initially, 79.4% were not frail, but this decreased to 60.1% at SM diagnosis, and 42.7% at 4-month follow-up. Consequently, the proportion of frail patients increased over the same period, with an 18.8% jump in mild frailty, 6.5% increase in moderately frail patients, and 11.5% increase in severely frail patients. Although status shifts continued from the 4-month to 2-year follow-up, the magnitude of change was relatively stable (Fig. 2A).

Rapid frailty progression early on in a patient’s disease course was also seen depending on the patient’s presentation. In patients with only 4-months data, 28% presented with moderate-severe frailty at metastatic diagnosis (Fig. 2B). This increased to 57% within 4 months, including 38% of previously not frail patients (*P* = 0.01, Fig. 2C). Only 19% of moderate-severe frailty patients at presentation improved to mild frailty with none exhibiting complete resolution.

A small proportion of patients had 1-year of follow-up data (*n* = 32); the vast majority of whom presented as not frail (62.5%). The proportion of patients with moderate-severe frailty increased rapidly, with 41% at 4 months, and 69% at 1 year (Fig. 2B). Similarly, 40% of not frail patients transitioned into moderate-severe frailty by 4 months (*P* = 0.26, Fig. 2C). Minimal improvement was seen in moderate-severe frailty patients, with 93.3% unchanged between 8 months and 1 year (*P* = 0.08, Fig. 2C).

Comparatively, patients with 2-years of follow-up data exhibited a slower trend toward frailty. While the proportion of frail patients increased over time, the percentage of moderate-severe frailty patients never exceeded 23% (Fig. 2B). Nearly half of patients (45%) remained not frail at 2-years post-SM diagnosis. Of note, 45.6% of mild frailty and 33.3% of moderate-severe frailty patients showed resolution of frailty early in their oncologic course, between 4 and 8 months (*P* < 0.001, Fig. 2C).

### Frailty status: contributing factors

**Fig. 3:**
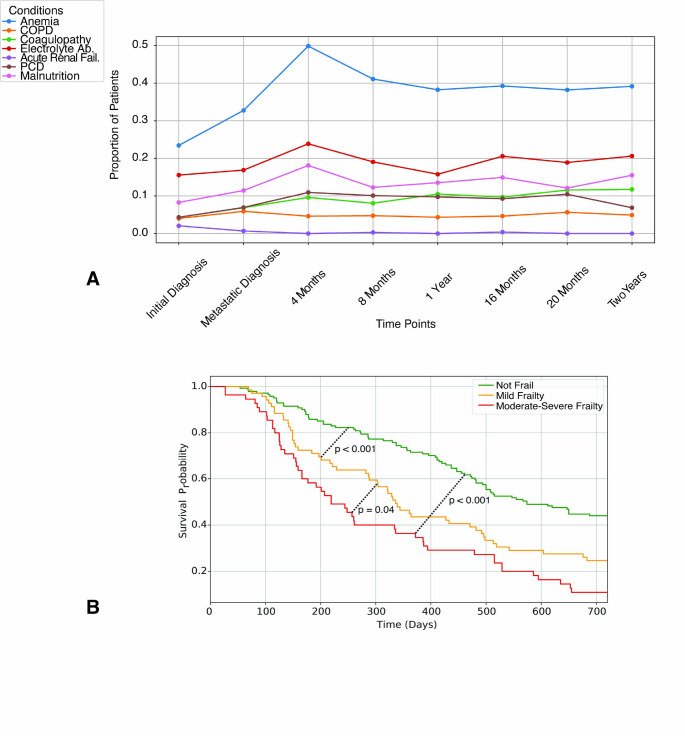
Contributing factors to frailty status and survival analysis. **A** Changes in MSTFI-7 components tracked over time show that anemia, electrolyte abnormalities, and malnutrition were most prevalent, peaking between metastatic diagnosis to 4-months postdiagnosis. These components stabilized by 8 months to 2 years, but over 20% of patients exhibited persistent anemia and electrolyte abnormalities (**B**) Kaplan-Meier curves indicate that higher frailty at presentation significantly reduces the probability of 2-year survival from metastatic diagnosis

Among all components of the MSTFI-7 frailty score, anemia (32.7%), electrolyte abnormalities (16.9%), and malnutrition (11.4%) were most common at SM diagnosis. All components, except COPD and renal failure, showed increased rates between metastatic diagnosis and 4-month follow-up, including 17.1% increase in anemia, 7% in electrolyte abnormalities, 6.7% in malnutrition, and 4.0% in PCD. While these conditions subsequently decreased in proportion from 4–8-month follow-up, anemia and electrolyte abnormalities remained at nearly 40% and 20%, respectively, of patients by the 2-year mark. COPD and renal failure were uncommon throughout the study duration (Fig. 3A).

Histological subtypes included frailty dynamics: In prostate cancer patients, the frailty score was 1.32 points higher at the 4-month only group (*P* < 0.01), but with no significant association with frailty scores at the 1-year only and 2-year groups (*P* = 0.5–0.8). In the 2-year group, NSCLC and other cancers both showed significant negative relative frailty scores of 0.67 points decrease (*P* = 0.01) and 0.74 points decrease (*p* < 0.01), respectively. Sarcoma and melanoma patients exhibited higher frailty associations at 4 months, with increases of 0.88 (*P* = 0.04) and 0.57 (*P* = 0.03), respectively, but showed lower associations in the 2-year group, with decreases of 0.48 points (*P* = 0.02) and 0.55 points (*P* = 0.01, Table 2).

Relative to baseline prostate, RCC (0.8 points; *P* < 0.001), NCSLC (1.25 points; *P* < 0.001), and other cancers (1.08 points; *P* < 0.001) exhibited higher frailty scores overall. In the 4-month patients, several histologic groups exhibited a lower combined frailty impact relative to prostate cancer, including RCC (0.34 points; *P* = 0.02), breast (1.28 points; *P* = 0.03), and other cancers (0.24 points; *P* < 0.01). NSCLC showed a higher relative association with frailty score in the 4-month group (0.09 points; *P* = 0.02). This positive association with NSCLC grew in the 2-year group (0.51 points; *P* = 0.01), and other cancers also showed a positive association with frailty relative to prostate (0.33 points; *P* < 0.01, Table 3).

**Table 2:** Liner Mixed-Effect model across histology

Fixed effects	Coefficient	z	*P*	95% CI
Prostate cancer				
Age	0.02	2.29	**0.02**	[0.002, 0.03]
Chemotherapy^1^	0.19	2.4	**0.01**	[0.04, 0.34]
Only 4-mo.	1.32	4.67	**0**	[0.002, 0.03]
Only 1-y.	0.06	0.22	0.83	[− 0.45, 0.57]
All 2-y.	0.09	2.5	0.5	[− 0.17, 0.35]
**RCC**				
Age	0.001	0.11	0.91	[− 0.02, 0.02]
Chemotherapy^1^	− 0.11	− 0.95	0.34	[− 0.35, 0.12]
Only 4-mo.	0.21	0.61	0.54	[− 0.47, 0.90]
Only 1-y.	0.31	0.47	0.64	[− 0.66, 0.31]
All 2-y.	− 0.18	− 0.72	0.47	[− 0.66, 0.31]
**NSCLC**				
Age	0.03	2.5	**0.01**	[0.01, 0.06]
Chemotherapy^1^	− 0.16	− 1.09	0.27	[− 0.46, 0.13]
Only 4-mo.	0.29	0.95	0.34	[−0.32, 0.90]
Only 1-y.	0.53	1.3	0.2	[− 0.27, 1.34]
All 2-y.	− 0.67	− 2.62	**0.01**	[− 1.16, −0.17]
**Sarcoma**				
Age	− 0.01	− 1.15	0.25	[− 0.02, 0.01]
Chemotherapy^1^	0.66	4.9	**0**	[0.4, 0.93]
Only 4-mo.	0.88	2.08	**0.04**	[0.05, 1.7]
Only 1-y.	0.04	0.14	0.89	[− 0.47, 0.55]
All 2-y.	− 0.48	− 2.25	**0.02**	[− 0.89, −0.06]
**Breast**				
Age	− 0.01	− 0.53	0.6	[− 0.03, 0.02]
Chemotherapy^1^	0.35	1.56	0.12	[− 0.01, 0.78]
Only 4-mo.	− 0.23	− 0.38	0.7	[− 1.42, 0.96]
All 2-y.	− 0.15	− 0.44	0.66	[− 0.83, 0.52]
**Melanoma**				
Age	0	− 0.15	0.88	[− 0.02, 0.02]
Chemotherapy^1^	0.45	2.1	**0.03**	[0.04, 0.87]
Only 4-mo.	0.57	2.2	**0.03**	[0.05, 1.09]
Only 1-y.	0.41	1.04	0.3	[− 0.36, 1.19]
All 2-y.	− 0.55	− 2.5	**0.01**	[− 0.98, −0.12]
**Other cancer**				
Age	− 0.004	− 0.53	0.6	[− 0.02, 0.01]
Chemotherapy^1^	0.33	3.17	**0.002**	[0.13, 0.54]
Only 4-mo.	0.03	0.1	0.92	[− 0.47, 0.52]
Only 1-y.	0.13	0.39	0.7	[− 0.54, 0.8]
All 2-y.	− 0.74	− 3.65	**0**	[− 1.14, − 0.34]

^1^ Any form of chemotherapy

*mo* month(s); NSCLC, non-small cell lung cancer; *P*, P value,* RCC*Renal cell carcinoma,* y* year(s), *z*, z score

The impact of age, available post-diagnostic time points, and chemotherapy treatment on frailty were assessed in the context of each histologic classification. Based on these variables, patients with the same histologic classification exhibited different frailty severities. Patients with fewer available time points, older age, and treatment with chemotherapy generally exhibited higher frailty scores

**Table 3:** Linear Mixed-Effect model on overall cohort

Fixed effects	Coefficient	z	*P*	95% CI
General cohort				
Baseline effects				
Age	0.001	0.432	0.666	[− 0.01, 0.01]
Only 4-mo	1.34	3.46	0.001	[0.58, 2.09]
All 2-y	− 0.01	− 0.03	0.98	[− 0.33, 0.32]
Chemotherapy^1^	0.2	1.83	0.07	[− 0.01, 0.41]
RCC	0.8	3.67	0	[0.37, 1.22]
NSCLC	1.25	5	0	[0.76, 1.74]
Sarcoma	0.39	1.59	0.11	[− 0.09, 0.87]
Breast	0.29	0.75	0.46	[− 0.47, 1.04]
Melanoma	0.58	1.7	0.09	[− 0.09, 1.25]
Other cancer	1.08	5.6	0	[0.70, 1.46]
**Histology interaction with patient group**				
RCC: only 4-mo	− 1.14	− 2.3	0.02	[− 2.12, −0.16]
NSCLC: only 4-mo	− 1.16	− 2.4	0.02	[− 2.1, −0.21]
Sarcoma: only 4-mo	− 0.46	− 0.7	0.5	[− 1.78, 0.86]
Breast: only 4-mo	− 1.57	− 2.25	0.03	[− 2.94, 0.11]
Melanoma: only 4-mo	− 1	− 1.77	0.08	[− 2.11, 0.11]
Other cancer: only 4-mo	− 1.32	− 3.01	0.003	[− 2.17, −0.46]
RCC: All 2-y	− 0.19	− 0.69	0.49	[− 0.72, 0.35]
NSCLC: All 2-y	− 0.74	− 2.51	0.01	[− 1.32, − 0.16]
Sarcoma: All 2-y	− 0.36	− 1.18	0.24	[− 0.97, 0.24]
Breast: All 2-y	− 0.17	− 0.46	0.64	[− 0.87, 0.54]
Melanoma: All 2-y	− 0.79	− 1.66	0.1	[− 1.72, 0.15]
Other cancer: All 2-y	− 0.75	− 3.26	0.001	[− 1.20, − 0.30]
**Histology interaction with treatment**				
RCC: chemo	−0.28	− 1.76	0.08	[− 0.59, 0.03]
NSCLC: chemo	− 0.39	− 2.34	0.02	[− 0.72, − 0.06]
Sarcoma: chemo	0.4	2.35	0.02	[0.07, 0.74]
Breast: chemo	0.16	0.55	0.58	[− 0.40, 0.71]
Melanoma: chemo	0.32	1.35	0.18	[− 0.15, 0.79]
Other cancer: chemo	0.14	1.01	0.32	[− 0.13, 0.41]

^1^ Any form of chemotherapy

Abbreviations: *mo* Month(s), *NSCLC* Non-small cell lung cancer, *P P* value, *RCC* Renal cell carcinoma, *y* year(s), *z* Z score

Baseline and multivariable associations of patient-specific variables with frailty were assessed across the cohort, using prostate cancer as reference histology. RCC, NSCLC, and other cancers generally showed higher frailty scores than prostate cancer. Individual analyses revealed higher frailty among prostate cancer patients in the 4-month-only group, but lower frailty relative to other histologies in the 2-year group. For treatment effects, chemotherapy was linked to higher frailty in sarcoma patients and lower frailty in NSCLC patients compared to prostate cancer.

At the individual histologic level, age and chemotherapeutic treatment were also reported. Age exhibited a mildly positive association with frailty score, as seen in prostate cancer (0.02 points; *P* = 0.02) and NSCLC (0.03 points; *P* = 0.01). Treatment with any kind of chemotherapy was positively associated with frailty in prostate cancer (0.19 points; *P* = 0.01), sarcoma (0.66 points; *P* < 0.01), melanoma (0.45 points; *P* = 0.03), and other cancers (0.33 points; *P* < 0.01).

The contribution of various patient and treatment factors were also assessed. Relative to prostate cancer, increased average frailty scores were seen in RCC (0.8 points; *P* < 0.01), NSCLC (1.25 points; *P* < 0.01), and other cancers (1.08 points; *P* < 0.01). The combined effect of NSCLC on frailty remained positive when considering chemotherapeutic treatment (0.86 points; *P* = 0.02). Regardless of histology, only having a 4-month post-diagnostic data point was associated with higher average frailty scores (1.34 points; *P* < 0.01).

### Survival outcomes

The early static assessment of frailty at SM diagnosis has long-term clinical implications. In comparing not frail versus mild frailty versus moderate-severe frailty, there was a strong decrease in patients’ 2-year survival probability proportional to the degree of frailty. These differences were statistically significant among all 3 groups (Fig. 3B).

## Discussion

### Frailty as a dynamic and time-sensitive parameter

To date, the literature surrounding frailty in MSD patients has primarily focused on the capacity of these indices to predict various postoperative outcomes and complications. However, there remains a paucity of data on frailty trajectory [11], which can provide a unique perspective on MSD patients’ oncologic course and identify actionable treatment opportunities. Our analysis shows that even in an index made up of a few phenotypic and comorbidity-based parameters, there is a clear time-varying change in patients’ frailty status Frailty exhibited substantial early progression, particularly between SM diagnosis and the 4-month follow-up. This may reflect the dual burden of systemic oncologic progression and therapies known to disproportionately affect physiological reserves [27–30]. Such rapid frailty progression is concerning, given its potential to exacerbate neurologic compromise in patients with SM. While frailty continued to progress up to the 2-year follow-up, the magnitude of change was relatively stable beyond the initial months post-SM diagnosis Patients with limited follow-up data exhibited more rapid frailty progression. Those with the longest follow-up data (2 years) showed a slower, more stable progression, highlighting the potential benefits of sustained oncological and supportive care [31]. Tailored interventions to mitigate frailty progression could improve clinical outcomes [24, 32].

### Contributing factors: what made these patients frail?

These findings have significant implications for clinical decision-making, underscoring the need for frailty assessments in treatment planning. Frailty has been identified as a strong predictor of postoperative complications, treatment tolerance, and overall survival in oncologic patients [33, 34]. Anemia, electrolyte abnormalities, and malnutrition were the most prevalent at SM diagnosis. While some improvement was observed, abnormalities remained elevated [6, 35–38]. Future studies should investigate the effect of mitigating these conditions on a patient’s frailty trajectory and survival [39].

### Influence of histology on frailty progression

Histological subtypes played a significant role in frailty dynamics, with certain cancers (e.g., sarcoma, melanoma, and NSCLC) showing higher associations with frailty at early follow-up and reduced contributions over longer follow-up time points. These findings are relevant in neuro-oncology, where histology often informs treatment decisions and prognosis [40, 41]. For example, patients with sarcoma or melanoma—a common cause of SM—may require closer monitoring for early frailty progression, particularly in the setting of aggressive treatments. Notably, prostate cancer, the most common histology in the cohort, demonstrated limited overall association with frailty. However, its higher frailty scores in the 4-month follow-up group suggest that cancers with an overall better prognosis and perhaps slower disease progression can significantly impact frailty early in the disease [42]. Additionally, chemotherapy was positively correlated with frailty in multiple cancer subtypes, underscoring the need to balance treatment intensity with supportive care measures to preserve functional status [42, 43] The contribution of patient-level factors, such as age and histology-specific frailty trends, further emphasize the need for individualized care strategies [44]. Increased frailty scores were observed in RCC, NSCLC, and other cancers relative to prostate cancer, with the impact of NSCLC on frailty being pronounced when chemotherapy was administered [45]. Patients with only 4 months of data exhibited significantly higher frailty scores, indicating a subset of patients with rapid disease progression and poorer prognoses.

### Implications for survival and prognostication

Frailty at SM diagnosis emerged as a critical predictor of survival, with moderate-severe correlating with significantly reduced 2-year survival probability [18, 33]. Patients presenting with moderate-severe had the lowest survival probability, followed by those with mild frailty, with not frail patients exhibiting the highest survival rates. For patients with SM, this finding aligns with the known impact of systemic disease burden on neurologic and overall outcomes [46, 47]. Integrating frailty assessments with neurologic evaluations, such as functional status or spinal instability scores, may improve prognostication and inform multidisciplinary treatment planning [48]. Beyond the dynamic changes in frailty, the early static assessment of frailty at metastatic diagnosis has significant implications for long-term survival The prognostic utility of frailty in this study is supported by studies in the literature which reported promising results using MSTFI to predict inpatient complications and mortality, particularly in prostate cancer patients [14]. Additionally, other evidence has suggested that MSTFI may be more sensitive to predicting postoperative complications in MSD patients as compared to other common indices such as the Charlson Comorbidity Index and Modified 5-item Frailty Index [49]. Notably, however, there remains uncertainty regarding the content validity and clinometric properties of comorbidity-based frailty indices such as MSTFI [11, 17]. While future studies will benefit from a focus on phenotypic frailty characteristics via functional assessments [2], these tools are currently challenging to scale to large cohort analyses or integrate into the clinical workflow Our findings ultimately highlight the multifactorial nature of frailty in MSD patients and underscore the need for integrated, personalized approaches to patient management. Future research should focus on targeted interventions to mitigate the impact of anemia, electrolyte imbalances, and malnutrition, as well as optimizing treatment regimens that minimize frailty progression. Incorporating frailty assessment into prognostic models and routine oncology care may enhance clinical decision-making and improve patient outcomes by facilitating tailored treatment interventions that increase survival and quality of life [4].

### Limitations

Given the retrospective design and variable follow-up intervals, bias may have been introduced, particularly in identifying frailty trends among high-mortality populations. While the 7-item MSFTI provides useful information with statistically significant correlations of biomarkers to frailty and survival, the original MSTFI was notably developed in a surgical cohort. To address possible issues with translation of this index to our nonsurgical SBRT cohort, we included only objective parameters with exclusion of surgical approach and hospitalization data. With regard to our patient characteristics, the limited representation of specific histologies, such as sarcoma and melanoma, may reduce the generalizability of findings. Prospective studies with standardized follow-up intervals and focused evaluations of neurologic outcomes are needed to validate these findings. Some patients may have not been evaluated or treated with SBRT, due to rapid disease progression or diagnosis at a more advanced stage; it is possible that these patients would have high frailty but were not captured in this study.

### Recommendations

Considering the significant impact of frailty on outcomes in SM patients, future research should prioritize the development of interventions aimed at mitigating frailty progression. Early management of anemia, electrolyte abnormalities, and malnutrition—conditions highly prevalent in this cohort—may offer avenues for reducing frailty and improving patient outcomes. The impact of frailty on physical function, and interventions to potentially modify a patients’ functional trajectory, should be explored to optimize patients’ quality of life. Ideal protocols should aim to integrate these parameters into patients’ electronic health records which would help identify frail patients and streamline initiation of appropriate intervention.

## Conclusion

This study underscored the dynamic nature of frailty in patients with vertebral metastases and its profound impact on survival. Early identification and management of frailty, particularly in high-risk histologies and time points, are integral for effective patient care. Tailored interventions targeting frailty-related conditions, coupled with ongoing neurologic monitoring, have the potential to improve quality of life and survival for this vulnerable population.

## Data Availability

The dataset generated during and/or analyzed during the current study are available from the corresponding author on reasonable request.
